# Gastro-duodenal fluid induced nuclear factor-*κappa*B activation and early pre-malignant alterations in murine hypopharyngeal mucosa

**DOI:** 10.18632/oncotarget.6824

**Published:** 2016-01-06

**Authors:** Dimitra P. Vageli, Manju L. Prasad, Clarence T. Sasaki

**Affiliations:** ^1^ Department of Surgery, Yale Larynx Laboratory Section of Otolaryngology, Yale School of Medicine, New Haven, CT, USA; ^2^ Pathology and of Surgery (Otolaryngology), Yale School of Medicine, New Haven, CT, USA

**Keywords:** hypopharyngeal cancer, NF-*κ*B, gastroduodenal reflux, bile acids, in vivo

## Abstract

We recently described the role of gastro-duodenal fluids (GDFs) in generating changes consistent with hypopharyngeal neoplasia through activation of NF-*κ*B pathway, using an *in vitro* model of human hypopharyngeal normal keratinocytes. Here, we further provide evidence that gastro-duodenal reflux is a risk factor for early pre-malignant alterations in hypopharyngeal mucosa (HM) related to an activated NF-*κ*B oncogenic pathway, using both an *in vitro* and a novel *in vivo* model of C57Bl/6J mice. Histological, immunohistochemical and automated quantitative analysis documents significant NF-*κ*B activation and early pre-malignant alterations in HM topically exposed to GDFs, compared to acid alone and other controls. Early pre-malignant histologic lesions exhibited increased Ki67, CK14 and ΔNp63, cell proliferation markers, changes of cell adhesion molecules, E-Cadherin and β-catenin, and STAT3 activation. The *in vivo* effect of NF-*κ*B activation is positively correlated with p-STAT3, Ki67, CK14 or β-catenin expression, while GDFs induce significant transcriptional activation of RELA(p65), bcl-2, TNF-α, STAT3, EGFR and wnt5A, *in vivo*. Our *in vivo* model demonstrates selectively activated NF-*κ*B in response to topically administrated GDFs, leading to early pre-malignant events in HM.

## INTRODUCTION

Hypopharyngeal squamous cell cancer is a head and neck cancer subtype with very high mortality rates even in early stage. The American Cancer Society estimates in 2015 approximately 3,400 new hypopharyngeal cancer cases, with poor prognosis. Among known risk factors, such as tobacco smoking and alcohol use, gastroduodenal reflux (GDRD), a variant of gastro-esophageal reflux disease (GERD), is also considered a potential risk factor as there is growing evidence that GDRD may have carcinogenic potential related to chronic effects of toxic gastroduodenal fluids (GDFs) on hypopharyngeal mucosa (HM) [1-3]. However, the precise role of GDRD in hypopharyngeal carcinogenesis and its etiopathological mechanisms remain unclear. Although the clinical prevalence and magnitude of gastro duodenal reflux is not fully known, there seems to be growing evidence that approximately 50-86 % of patients with GERD present with mixed gastric and duodenal fluids (bile acids) in their refluxates [4, 5]. There is further evidence that extraesophageal bile acids found in the upper aero digestive tracts of patients are now linked to advanced inflammatory lung disease [6]. It is also well known that components of gastro-duodenal fluids, including conjugated bile acids and unconjugated deoxycholic acid (DCA) contribute to the development of Barrett's oesophagus and oesophageal adenocarcinoma, while DNA damage induced by bile acids on Barrett's cells correlates with constitutive activation of NF-*κ*B [7-16].

We recently described NF-*κ*B as a mechanistic link between GDFs stimulus-response in human hypopharyngeal normal keratinocytes and hypopharyngeal neoplasia, *in vitro* [17]. We also identified *in vitro* a GDFs-induced mRNA phenotype, which is commonly found in a human hypopharyngeal cancer cell line, including oncogenic STAT-3, TNF-α, ΔNp63 and WNT5A genes that have been previously linked to head and neck squamous cell carcinomas (HNSCC) [18-28]. The nuclear factor kappa B (NF-*κ*B) is a transcriptional factor complex consisting of homo- and heterodimers of five members of the Rel family [RelA (p65), RelB, c-Rel, NF-κba;B1 (p50/p105), NF-κba;B2 (p52/p100)] that has been considered a link between inflammation and cancer [12].

In the current study we will explore the effect of GDFs on murine HM in activating the NF-*κ*B pathway to induce molecular and/or histopathological alterations, linked to malignant transformation. We will use bile salts at molar concentrations close to physiological [11, 29, 30], but pharmacological non-toxic doses of bile acids, DCA and chenodeoxycholic acid (CDCA), that are topically applied to murine HM. First we will demonstrate that the chronic effect of GDFs on murine hypopharyngeal primary cells (MHPCs) *in vitro* can induce NF-*κ*B activation and bcl-2 overexpression. Then, we will provide evidence, using a mouse model that the chronic effect of GDFs on HM, is a causative factor for early pre-malignant histopathologic events, related to NF-*κ*B activation, overexpression of cell proliferation markers, activation of ΔNp63 and STAT3 oncogenes, and epithelial mesenchymal transition (EMT). We will further show that GDFs treated HM *in vivo* exhibits significant transcriptional activation of RELA(p65), bcl-2, EGFR, WNT5A, TNF-α and STAT3, as we similarly identified in human hypopharyngeal normal keratinocytes, *in vitro* [17].

## RESULTS

### The *in vitro* effect of GDFs on MHPCs induces NF-κB activation and bcl-2 overexpression

**Figure 1:**
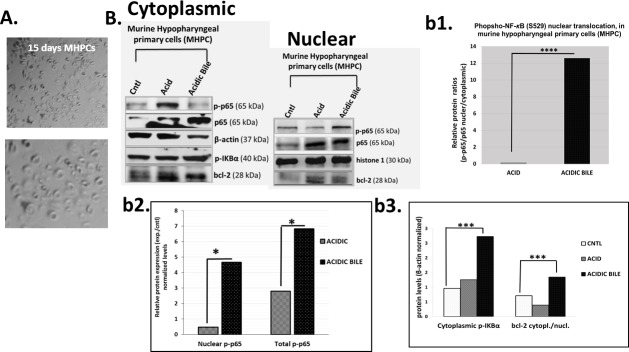
Gastro-duodenal fluids (GDFs) induced NF-*κ*B activation and bcl-2 overexpression in murine hypopharyngeal primary cells (MHPCs), *in vitro* **A.** Murine Hypopharyngeal Primary Cells (MHPCs) cultured in KGM-2 SFM medium for 15 days after dissociation of cells from hypopharyngeal tissue of C57Bl6J mice. **B.** Western blot analysis for total NF-*κ*B (p65), p-NF-*κ*B (p-p65), p-IKBα and bcl-2 protein levels (b1) nuclear p-NF-*κ*B (p-p65 S529) translocation value (nuclear/cytoplasmic p-p65/p65 ratios); (b2) nuclear and total p-NF-*κ*B (p-p65) experimental to untreated control (exp./control) expression ratios; (b3) cytoplasmic p-IKBα (Ser32/36) and cytoplasmic/nuclear bcl-2 levels (*p < 0.05; ***p < 0.0005,****p < 0.0005; by z-test).

The repetitive exposure of MHPCs to acidic-bile resulted in a higher nuclear p-NF-*κ*B (p65 S529) translocation value (nuclear/total p-p65/p65) and significantly higher cytoplasmic p-IKB-α levels, compared to acid or neutral controls (*P* < 0.05) (Figure 1B-b1,2). Although an acidic environment contributes to NF-*κ*B activation, MHPCs treated by acidic-bile fluids exhibited higher total and nuclear p-NF-*κ*B relative expression (experimental/neutral-control) compared to acid alone (*P* < 0.05) with significantly higher cytoplasmic/nuclear bcl-2 ratios, compared to neutral-control (*P* < 0.05) (Figure 1B-b3).

A statistically significant Pearson's correlation was found between cytoplasmic bcl-2 and activated p-NF-*κ*B levels (*r* = 0.9780) (*P* < 0.05), as well as between cytoplasmic bcl-2 and p-IKB-α levels (*r* = 0.9744, *P* < 0.05), in treated MHPCs.

Together, our data indicate that the effect of GFs on MHPCs, *in vitro*, especially the combination of acid and bile salts, resulted in NF-*κ*B activation that was related to significant accumulation of bcl-2 to the cytoplasm, suggesting anti-apoptotic function [37]. The results are consistent with our previously reported observations in HHKs [17].

### The *in vivo* effect of GDFs induces pre-malignant alterations in murine HM

**Figure 2:**
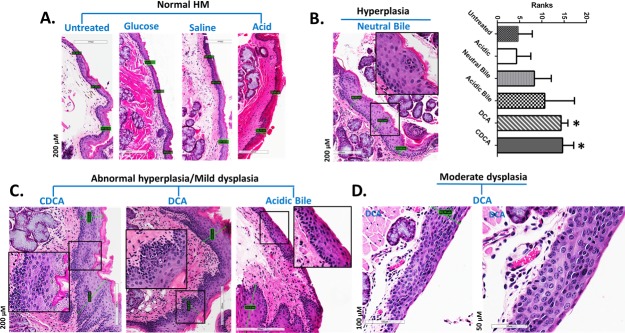
GDFs-induced premalignant lesions in murine hypopharyngeal mucosa of C57BL6J mice (H&E staining) **A.** Normal HM: keratinized stratified squamous epithelium/single layer of basal cells. **B.** Hyperplastic HM: thickness of stratified epithelium and hyperchromatic or pleiomorphic basal cells expanding in the stratum spinosum. Columns of graph created be GrapPad software, correspond to thickness means of HM under different treatment. The upper line of column indicate the highest measurement. (*p < 0.05; ANOVA, Kruskal-Wallis test; Graph Pad Prism 6.0). **C.** Abnormal hyperplastic/mild dysplastic HM: thickness of stratified epithelium, hyperchromatic or pleiomorphic basal cells expanding in the stratum spinosum, nuclear atypia into the middle third of the mucosa. **D.** Moderate dysplastic HM: full-thickness, nuclear hyperchromatism, high degree of basal layer expansion, large cells with increase of nuclear to cytoplasm ratios near the surface, and/or loss of cells polarity large.

The chronic effect of gastroduodenal fluids (GDFs) on murine HM, *in vivo*, resulted in early pre-malignant lesions of HM [31, 32, 34, 35, 38] (Figure 2). Microscopic examination of H&E-stained hypopharyngeal mucosa, revealed hyperplastic and dysplastic changes, compared to normal HM (Figure 2). In contrast, HM from mice exposed to acid alone, glucose or saline resulted in no histologic change (Figure 2A). Specifically, we observed that the HM exposed to bile salts at neutral or acidic pH, DCA or CDCA exhibited hyperplastic lesions with significantly increased thickness compared to normal untreated-HM (1.8-4.6 X normal) (*p* < 0.05; Kruskal-Wallis test) (Figure 2B). Abnormal hyperplasia or mild dysplasia was identified in CDCA, DCA and acidic-bile treated-HM (Figure 2C), while moderate dysplastic lesions were identified in DCA treated-HM (Figure 2D).

Table 2 presents the percentages of mice that exhibited histopathological alterations by treatment category. There were no histological signs of local treatment toxicity.

**Table 1:** Mouse genes (targets and GAPDH) and their detected transcripts, analyzed by real time qPCR, in murine hypopharyngeal mucosa

Gene (mouse)	Detected transcripts	Amplicon length (bp)
GAPDH	NM_008084	144
	NM_001289726	
RELA	NM_009045	82
STAT3	NM_011486	99
	NM_213659	
	NM_213660	
WNT5A	NM_001256224	130
	NM_009524	
BCL-2	NM_009741	80
TNFA	NM_013693	112
	NM_001278601	
EGFR	NM_007912	68
	NM_207655	

**Table 2:** Percentage (%) of C57BL/6J mice exhibited histopathological alterations of hypopharyngeal mucosa (HM) and mucosa compartments exhibited molecular alterations of NF-κB, ΔNp63, and cell-proliferation markers Ki67 and CK14

	Normal* HM	Acidictreated HM	Neutral Biletreated HM	Acidic Biletreated HM	DCA treated HM	CDCA treated HM
**C57Bl67 mice** surv./total	5/5	3/5	5/5	4/5	5/5	4/5
**Hyperplasia/Dysplasia** % (obser./surv.) mice	**0%** (0/5)	**0%** (0/3)	**100%** (5/5)	**75%** (3/4)	**100%** (5/5)	**50%** (2/4)
**Molecular alterations** (mucosa/cell compartments)					
**p-NF-*κ*B /** nuclei	-	BL/PL	BL/PL/SL	BL/PL/SL	BL/PL/SL	BL/PL/SL
**ΔNp63** / nuclei	BL	BL/PL	BL	BL/PL/SL	BL/PL/SL	BL/PL/SL
**Ki67** / nuclei	BL	BL	BL/PL/SL	BL/PL/SL	BL/PL/SL	BL/PL/SL
**CK14** / cytoplasmic	BL/KL	BL/KL	Whole thickness	Whole thickness	Whole thickness	Whole thickness

* Untreated HM; BL: basal layer; PL: parabasal layer; SL: suprabasal layer; KL: keratinized layer; observ.: observed; surv.: survived

### The *in vivo* effect of GDFs induces NF-κB activation, and correlates with histologic pre-malignant alterations in HM

**Figure 3:**
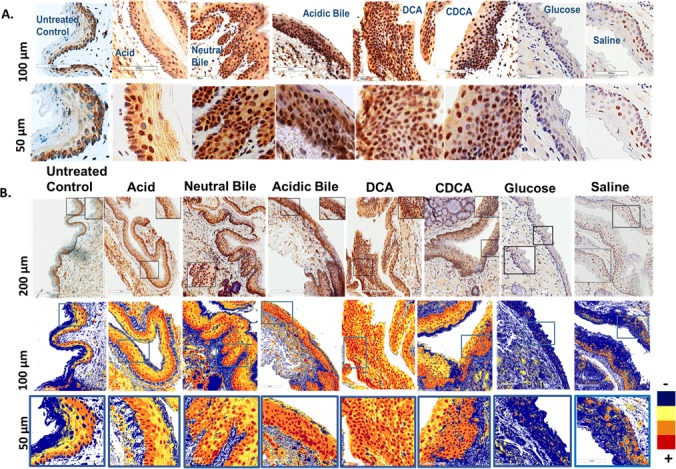
*In vivo* GDFs-induced NF-*κ*B activation in murine hypopharyngeal mucosa (HM) **A.** Immunohistochemical analysis (IHC) for p-NF-*κ*B (p65 S536) (from left to right): control (normal) untreated-HM, cytoplasmic staining; acid alone treated-HM, nuclear or cytoplasmic staining mainly of cells of basal layer and weak cytoplasmic staining of suprabasal layers; hyperplastic/dysplastic neutral-bile, acidic-bile, DCA and CDCA treated-HM, intense nuclear and cytoplasmic staining of cells of basal and suprabasal layers; glucose and saline treated-HM, cytoplasmic staining sporadically of a few basal/parabasal cells. **B.** Image analysis algorithm(s) (red, nuclear positive staining of p-NF-*κ*B; orange, intense positive cytoplasmic staining of p-NF-*κ*B; yellow, weak cytoplasmic staining of p-NF-*κ*B; blue, negative p-NF-*κ*B staining).

The microscopic examination of GDFs treated-HM, and particularly at sites of pre-malignant change, revealed a significant activation of NF-*κ*B (p65 S536) throughout its thickness, relative to normal (untreated control, glucose or saline treated controls) (Figure 3A). We found that either neutral bile or acidic bile mixtures, and bile acid components, DCA or CDCA, induced elevated NF-*κ*B activation, producing an intense p-NF-*κ*B nuclear staining of cells in basal and suprabasal layers (Figure 3B). In contrast, the untreated-HM did not exhibit NF-*κ*B activation by virtue of negative nuclear p-NF-*κ*B (p65 S536) staining. Acid alone induced less intense and less extensive NF-*κ*B activation in HM limited to the basal layer, compared to the GDFs, and particularly to acidic-bile. Finally, we found that the glucose and saline treated controls exhibited low levels of NF-*κ*B activation in few and sporadic cells, and both were considered negative for NF-*κ*B activation.

### The GDFs-induced NF-κB activation is related to molecular alterations linked to premalignant transformation of HM

**Figure 4:**
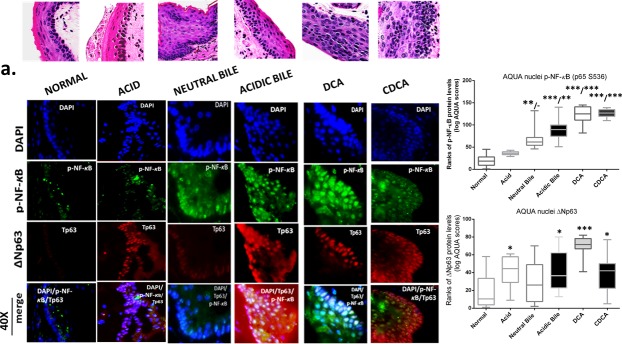

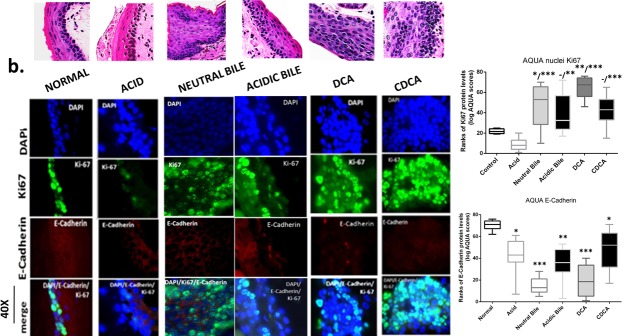

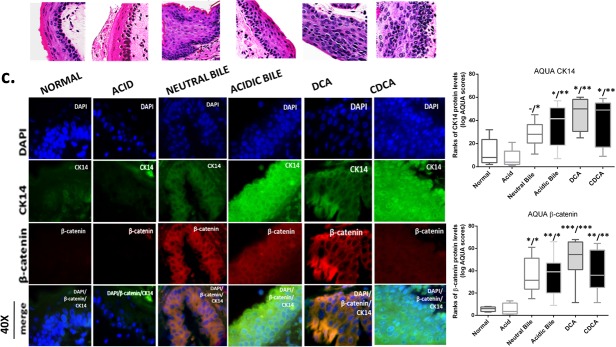

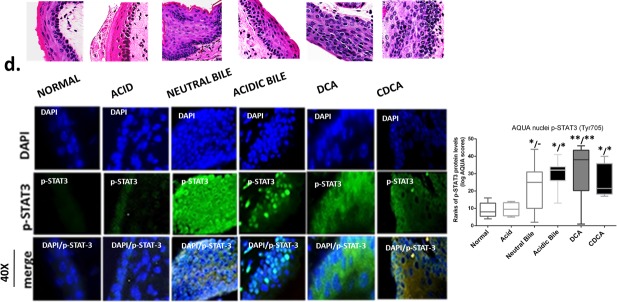
Molecular alterations underlying GDFs induced histopathological alterations of murine hypopharyngeal mucosa (HM), *in vivo*, linked to p-NF-κB activation, increased cell proliferation, cell-cell adhesion changes, and STAT3 activation Immunofluorescence (IF) staining and automated quantitative analysis (AQUA) for (a) p-NF-*κ*B (p65 S536) (green) and ΔNp63 (red); (b) Ki67 (green) and E-cadherin (red); (c) CK14 (green) and ϐ-catenin (red); and (d) p-STAT3 (Tyr705) (green) (from left to right): Normal untreated-HM, acid alone treated-HM, and hyperplastic/dysplastic neutral-bile, acidic-bile, DCA, and CDCA treated-HM derived from C57Bl6J mice. DAPI (blue) was used for nuclei staining. (DyLight^®^488 for green and DyLight^®^549 for red). The boxplots, represent the means ranks. The upper line indicates the highest value, the lower line the lowest value and the middle line the mean of AQUA normalized quantities of each variable. Statistically significant difference between AQUA-score means is indicated for GDFs *vs*. acid (left) and GDFs or acid treated *vs*. normal-untreated (right) (*p < 0.01; **p < 0.001;***p < 0.0001; ONE-WAY ANOVA, Kruskal-Wallis and Dunn's multiple comparison tests; Graph Pad Prism 6.0).

**Figure 5:**
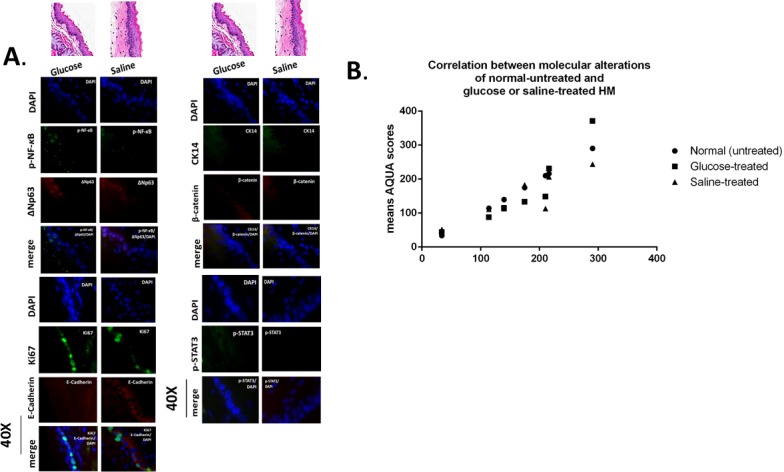
Murine hypopharyngeal mucosa (HM) exposed to glucose or saline present normal histological features and immunofluorescence (IF) staining patterns similar to normal-untreated HM **A.** IF staining for (a) p-NF-*κ*B (p65 S536) (green) and ΔNp63 (red); (b) Ki67 (green) and E-Cadherin (red); (c) CK14 (green) and ϐ-catenin (red); and (d) p-STAT3 (Tyr705) (green). DAPI (blue) was used for nuclei staining. (DyLight^®^488 for green and DyLight^®^549 for red). **B.** Graph presents linear correlation between AQUA means of glucose or saline-treated HM and normal untreated-HM (Paired-t test, GraphPad Prism 6.0).

The *in vivo* effect of GDFs on murine HM, induced molecular alterations related to NF-*κ*B activation and malignant transformation of hypopharygeal mucosa, by increased expression of cell proliferation markers Ki67 and CK14, changes of cell-cell adhesion molecules E-cadherin and β-catenin, as well as elevated levels of ΔNp63 and activation of p-STAT3 (Tyr705) oncogene, compared to normal untreated-HM or acid alone treated tissues (Figure 4). The glucose and saline treated controls demonstrated IF-staining patterns that were similar to normal untreated-HM (Figure 5). Using AQUA analysis, we found that the GDFs treated-HM exhibited significantly higher total protein levels of the analyzed molecules (p-NF-*κ*B, Ki67, ΔNp63, p-STAT3, CK14 and β-catenin), compared to untreated-HM (*P* < 0.00274) or acid alone (*P* < 0.0055) (by *Friedman test*). In contrast, the GDFs treated-HM demonstrated a significant decrease of E-cadherin expression, relative to untreated-HM (*P* < 0.05). Interestingly, we observed that the AQUA means of the analyzed markers (nuclear p-NF-*κ*B, Ki67, ΔNp63, p-STAT3 and total CK14 and β-catenin) exhibited a significant linear correlation between glucose treated and untreated-HM (*r* = 0.9417, *P* < 0.0039), and between saline treated and untreated-HM (*r =* 0.8824, *P* < 0.0085) (Paired t-test, Prism) (Figure 5).

Our results from IF staining and statistical analysis of AQUA-scores (ONE-WAY ANOVA, Kruskal-Wallis, Dunn's multiple tests) of each molecular marker, in GDFs treated-HM and controls (Figure 4), are described as follows:

#### GDFs-induced p-NF-κB and ΔNp63 activation

The IF staining of p-NF-*κ*B provided in Figure 4A, confirmed our results from chromogenic-staining (Figure 3). We observed that neutral-bile, DCA and particularly acidic-bile treated-HM, produced an intense p-NF-*κ*B (p65 S536) (green) nuclear staining of the basal layer that was also expanded to the suprabasal layers, particularly at pre-malignant sites. In contrast, normal untreated-HM exhibited an intense cytoplasmic p-NF-*κ*B staining limited to the basal layer. Acid alone treated-HM exhibited a normal histologic structure, with nuclear p-NF-*κ*B staining limited to the basal and immediate suprabasal layers. AQUA analysis revealed significantly higher nuclear p-NF-*κ*B means of AQUA scores (AQUA-means) in the GDFs treated-HM, relative to untreated-control (acidic-bile, DCA, CDCA treated *vs*. untreated, *P* < 0.0001; neutral-bile *vs*. untreated, *P* < 0.001) or acid alone treated-HM (DCA, CDCA *vs*. acid, *P* < 0.0001; acidic-bile *vs*. acid, *P* < 0.001).

We also observed that the same acidic-bile, DCA or CDCA treated-HM, exhibited an intense nuclear ΔNp63 expression in the basal and parabasal layers, which expanded in several sites to the suprabasal layers (Figure 4A). In contrast, normal untreated-HM provided a weak ΔNp63 expression, limited to cells of the basal layer. Similarly, acid alone treated-HM demonstrated nuclear ΔNp63 expression that was limited to cells of the basal layer and in a few cells of the parabasal layer. AQUA analysis revealed significantly higher nuclear ΔNp63 AQUA-means in the GDFs treated-HM compared to untreated-control (DCA treated *vs*. untreated, *P* < 0.0001; acidic-bile, CDCA treated *vs*. untreated, *P* < 0.01), as well as in HM exposed to acid alone (*P* < 0.01), relative to untreated-control.

#### GDFs-induced increased Ki67 and decreased E-cadherin levels

We found an elevated and expanded Ki67 (green) nuclear expression in GDFs treated-HM (Figure 4B). Specifically, abnormal hyperplastic CDCA treated-HM, demonstrated a significant expansion of Ki67 staining in the parabasal and suprabasal layers, compared to normal untreated-HM, which produced a few positive cells in the basal layer. Also, acid alone treated-HM presented a weak Ki67 staining that was limited in few cells of the basal layer, which was much weaker compared to untreated-HM. AQUA analysis revealed significantly higher nuclear Ki67 AQUA-means in the GDFs treated-HM, which featured pre-malignant alterations, relative to normal untreated-HM (DCA and neutral-bile treated *vs*. untreated *P* < 0.001 and *P* < 0.01, respectively) or acid alone treated-HM (DCA, CDCA, neutral bile *vs*. acid alone, *P* < 0.0001; acidic-bile *vs*. acid alone, *P* < 0.001).

Moreover, the same GDFs treated-HM exhibited a less intense E-cadherin (red) staining compared to normal untreated-HM, in which E-cadherin was intense within its entire thickness (Figure 4B). AQUA analysis revealed significant decrease of E-cadherin AQUA-means in the GDFs treated, compared to untreated HM (DCA, neutral-bile treated *vs*. untreated, *P* < 0.0001; acidic-bile and CDCA treated *vs*. untreated, *P* < 0.001 and *P* < 0.01, respectively). Also, the acid alone treated-HM exhibited lower E-cadherin levels relative to untreated-HM (*P* < 0.01).

#### GDFs-induced increased CK14 and β-catenin levels

We demonstrate an extended CK14 (green) expression in the entire thickness of GDFs treated-HM, compared to untreated or acid alone treated-HM, in which CK14 was limited to the basal layer (or to the keratinized layer, since murine HM is often keratinized) (Figure 4C). AQUA analysis showed a significant increase of CK14 AQUA-means in the GDFs treated-HM relative to untreated-HM (acidic-bile, DCA, CDCA treated *vs*. untreated *P* < 0.01) or acid alone treated-HM (acidic-bile, DCA, CDCA *vs*. acid alone, *P* < 0.001; neutral-bile *vs*. acid alone, *P* < 0.01) (Figure 4C).

Additionally, the GDFs treated-HM, particularly neutral-bile, acidic-bile or DCA treated-HM presented an intense staining of β-catenin (red), compared to the normal untreated-HM or acid alone treated-HM presented a weak β-catenin staining (Figure 4C). AQUA analysis revealed significantly higher β-catenin AQUA-means in the GDFs treated-HM relative to untreated-HM (DCA, CDCA, acidic-bile treated *vs*. untreated, *P* < 0.001; neutral-bile treated *vs*. untreated, *P* < 0.01) or acid alone treated-HM (DCA, CDCA *vs*. acid alone, *P* < 0.001; acidic-bile, neutral-bile *vs*. acid alone, *P* < 0.01) (Figure 4C).

#### GDFs-induced STAT3 activation

We identified an intense p-STAT3 (green) nuclear staining in the GDFs treated-HM, and particularly in acidic-bile treated HM with pre-malignant alterations, indicating activation of STAT3. In contrast, the normal untreated-HM was negative for STAT3 activation, while acid alone treated-HM produced a weak p-STAT3 nuclear staining (Figure 4D). AQUA analysis revealed significantly higher nuclear p-STAT3 (Tyr705) AQUA-means in the GDFs treated-HM, relative to untreated-HM (DCA treated *vs*. untreated *P* < 0.001; acidic-bile, neutral-bile, CDCA treated *vs*. untreated, *P* < 0.01) or acid alone treated-HM (DCA *vs*. acid alone, *P* < 0.001; acidic-bile, CDCA *vs*. acid alone, *P* < 0.01) (Figure 4D).

### Correlations among p-NF-κB, Ki67, ΔNp63, CK14, β-catenin and E-cadherin molecular alterations induced by GDFs in murine HM

**Figure 6:**
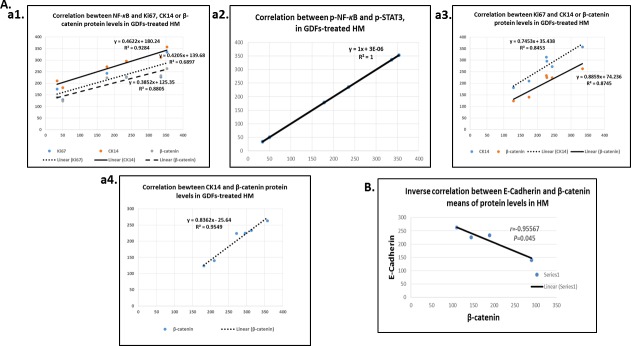
Diagrams show significant linear correlations by Pearson **A.**, among altered protein expression levels upon GDFs treatment in murine HM; (a1) between p-NF-*κ*B and Ki67, CK14 or β-catenin; (a2) between p-NF-*κ*B and p-STAT3; (a3) between Ki67 and CK14 or β-catenin; and (a4) between CK14 and β-catenin. **B.** Diagram depicting an inverse correlation between E-cadherin and β-catenin levels.

*Pearson* analysis revealed significantly positive relationships among the underlying molecular alterations of pre-malignant murine HM affected by GDFs (Graph Pad Prism 6.0). We identified positive Pearson's correlations between the expression levels (AQUA means) of activated NF-*κ*B and Ki67 (*r* = 0.714, *P* < 0.047), CK14 (*r =* 0.963, *P* < 0.0020), β-catenin (*r =* 0.938, *P* < 0.0056) or activated p-STAT3 (*r* = 0.8785, *P* < 0.0231) (Figure 6A-a1 & a2). Additionally, a positive correlation was identified between the expression of the p-NF-*κ*B and ΔNp63 (*r* = 0.5838) although was not statistically significant.

We also observed a strong positive correlation coefficient between the expression levels of Ki67 and CK14 (*r* = 0.938, *P* < 0.0062), Ki67 and β-catenin (*r* = 0.919, *P* < 0.0095), and CK14 and β-catenin (*r* = 0.9777, *P* < 0.0008) (Figure 6A-a3 & a4). In contrast, we observed a significant inverted correlation between the expression of the E-cadherin and β-catenin (*r* = −0.95567, *P* < 0.045; by *Pearson*) (Figure 6B), particularly noted in the pre-malignant neutral-bile and DCA treated-HM (*P* < 0.001, χ2-test).

### GDFs-induced transcriptional activation of NF-κB related oncogenic pathways in murine HM, *in vivo*

**Figure 7:**
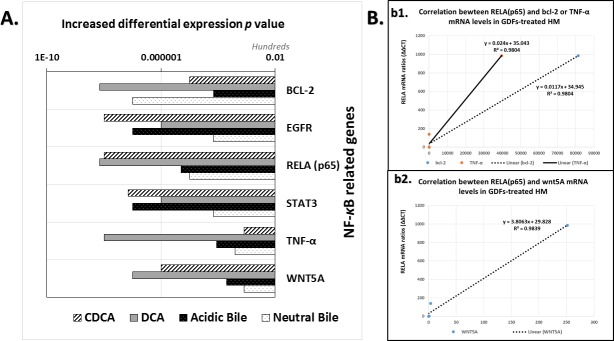
GDFs-induced transcriptional activation of NF-*κ*B related oncogenic pathways in murine hypopharyngeal mucosa (HM), *in vivo* **A.** Graph presents the increased differential mRNA expression of NF-κB related oncogenic pathways between GDFs treated and untreated-control murine HM (*p* values by z-test). (GAPDH was used as housekeeping gene to normalize mRNA expression values of each target gene). **B.** Diagrams depicting strong linear correlation between RELA(p65) and bcl-2, TNF-α (b1) or WNT5A (b2), mRNA relative expression ratios (GDFs treated/untreated control) in murine HM (by *Pearson*).

We observed that the *in vivo* effect of GDFs on murine HM resulted in transcriptional activation of NF-*κ*B oncogenic pathways, as Sasaki et al., demonstrated *in vitro* on HHKs [17]. Our analysis, by real time qPCR, showed a significant overexpression of NF-*κ*B transcription factor, RELA (p65), and NF-*κ*B related genes, bcl-2, EGFR, WNT5A, TNF-α, and STAT3, in GDFs-treated HM, and particularly with DCA (*P* < 0.0021), CDCA (*P* < 0.0076) and acidic-bile (*P* < 0.0423) treated-HM, compared to untreated-control (by Friedman test, Dunn's multiple comparisons). The differential expression (*p* values) of each target gene and group of treatment is presented in Figure 7A.

The DCA treated-HM produced the most accelerated transcriptional levels of the analyzed genes. Notable, the acidic-bile and CDCA treated-HM exhibited the highest expression ratios of the STAT3 and EGFR mRNAs relative to untreated-control, and higher WNT5A and RELA (p65) mRNA levels compared to neutral-bile treated-HM.

Moreover, the real time qPCR analysis revealed the follows:

(i) Each studied gene, exhibited a significant overexpression in the GDFs treated-HM relative to untreated-control, while the NF-κB transcription factor, RELA(p65), the EGFR, the STAT3 oncogene, and the anti-apoptotic bcl-2, were among the most overexpressed (Figure 7B). (ii) The NF-κB transcription factor (p65) presented similar expression pattern at the mRNA level [RELA(p65)], quantified by real-time PCR, and at protein levels (p-p65, S536), obtained by AQUA, compared to untreated control (Figures 4A and 7A). (iii) The DCA and neutral-bile treated-HM exhibited significantly higher mRNA levels of the anti-apoptotic bcl-2 compared to CDCA and acidic-bile treated-HM (*P* < 0.000, by χ2-test), while the same groups had also been found by AQUA, to express the highest cell proliferation Ki67 levels and β-catenin/E-cadherin expression ratios (Figures 4B and 7A).
- Moreover, we found a strongly positive Pearson's correlation between mRNA levels of NF-*κ*B transcriptional factor, RELA(p65) and (i) anti-apoptotic bcl-2 (*r =* 0.990113, *P* = 0.0012), (ii) TNF-α (*r* = 0.990385, *P* = 0.0011) or (iii) oncogenic WNT5A (*r* = 0.992301, *P* = 0.0008), in GDFs affected murine mucosa (by *Pearson*) (Figure 7B).

## DISCUSSION

Our prior *in vitro* study in human hypopharyngeal normal keratinocytes (HHKs) supported the role of gastro-duodenal fluids (GDFs) induced NF-*κ*B pathway in hypopharyngeal carcinogenesis [17]. Here we explored the crosstalk between GDFs stimulus-response and tumorigenic processes in murine hypopharyngeal mucosa through activation of NF-*κ*B pathway, using both an *in vitro* model of murine hypopharyngeal primary cells (MHPCs), and a novel *in vivo* mouse model. Our findings provide evidence that bile reflux is a causative risk factor of pre-malignant alterations in hypopharyngeal mucosa, related to NF-*κ*B pathway, in line with established studies involving GDFs (bile acids) in the development of Barrett's esophagus, also considered a precancerous condition [39], *via* NF-*κ*B pathways [7-9].

In our animal model, we provide evidence that the constitutive stimulation of murine HM by GDFs, even in a short period of 45 days, is capable of activating a tumorigenic process. Our data supporting the effect of GDFs are specific, since only mixtures of bile acids or bile acid components DCA and CDCA, but not acid alone or other factors, such as concentrated glucose, induce molecular alterations and early pre-malignant lesions, such as abnormal hyperplasia or dysplasia, previously linked to high risk of carcinomatous transformation of upper aerodigestive tract squamous epithelium [34, 38, 40].

We present by IHC analysis that NF-*κ*B may be an important mediator in GDFs-induced inflammatory to early-premalignant events in HM, in ways similarly linked to Barrett's esophagus [10]. Evidence of bile acid tumorigenic potential, *via* NF-*κ*B, is based on the advanced NF-*κ*B activation identified in HM exposed to bile acids or their components DCA and CDCA, at sites with abnormal histopathological features. In contrast normal mucosa and mucosa treated with glucose, were negative for NF-*κ*B activation, in line with our previous *in vitro* findings in HKKs [17]. The magnitude of NF-*κ*B activation in GDFs treated-HM is demonstrated by AQUA analysis. Our findings from both IHC and AQUA analysis indicate a specific effect of acid and bile combination on significant NF-*κ*B activation in HM, compared to acid alone, again in line with our prior *in vitro* findings [17]. Notably, our findings describe that the combination of bile and acid is be more effective in inducing activation of NF-*κ*B than neutral bile.

We provide evidence of NF-*κ*B role as a mechanistic link between GDFs effect and early tumorigenic processes in HM, by presenting a significant correlation between the advanced NF-*κ*B activation and underlying alterations of cell proliferation and epithelial to mesenchymal transition (EMT) markers of oncogenic pathways. Notably, there was no evidence of cell proliferation, cell-cell adhesion changes in normal mucosa or mucosa treated by acid alone, saline or glucose.

Cell proliferation markers (Ki67 and CK14) have been considered to determine molecular alterations at early pre-malignant mucosa of the upper aero-digestive tract [41-44]. Our data showed that GDFs treated mucosa, particularly at pre-malignant sites, exhibited increase of cell proliferation rates, indicated by an elevated and expanded expression of Ki67 and CK14. On the other hand, acid alone treated HM showed both low NF-*κ*B activation and Ki67 and CK14 expression, implying molecular changes that correspond to reduced cell proliferation rates or apoptosis [45].

Our novel *in vivo* data support an EMT process, in GDFs treated-HM, indicated by significant changes in cell-cell adhesion molecules, such as β-catenin and E-cadherin, previously linked to oral premalignant lesions, while the latter has been related to laryngopharyngeal reflux [46-48]. We present an inverted expression between E-Catherin and β-catenin proteins in GDFs treated-HM, compared to normal untreated mucosa or mucosa treated by acid alone, implying possible alterations of tight cell adhesions to a more mobile and loosely mesenchymal phenotype that has been previously identified as precursor to cancer [18, 47, 48]. We are able to show that repetitive exposure of murine HM to GDFs, resulted in molecular changes linked to EMT through NF-*κ*B activation, by associating a significant positive correlation between β-catenin and advanced NF-*κ*B activation in HM treated by bile salts.

The ΔNp63 is considered oncogenic and an important regulating factor of epithelial cell proliferation in HNSCCs [49]. Our *in vivo* findings demonstrate ΔNp63 as a major index of the GDFs-induced tumorigenic potential, which appears particularly related to acid and bile salts combination, *via* NF-*κ*B. Our data are in agreement with our prior *in vitro* data from HHKs [17] and with previous studies supporting the ΔNp63/NF-*κ*B interaction in cell proliferation processes of epithelial cells [22]. On the other hand, the relative increase ΔNp63 expression under acid alone exposure does not appear to be related to cell proliferation but rather to apoptotic processes [50].

Moreover, we demonstrate the activation of oncogenic STAT3 on murine HM as linked to head and neck carcinogenesis [26-28]. Interestingly, STAT3 activation is significantly intense at sites of early pre-malignant HM affected by acidic-bile or DCA. We also show a linear correlation between STAT3 and NF-*κ*B activation that is also demonstrated at transcriptional levels, in line with our prior *in vitro* study in HHKs and other studies [17, 27, 28].

Our novel *in vivo* findings demonstrate a significant overexpression of NF-*κ*B related oncogenic pathways providing further evidence of NF-*κ*B role as a mechanistic link between GDFs induced stimulus-response *in vivo* murine HM, in line with our prior *in vitro* study in HHKs [17]. We assert that GDFs, affect HM by inducing significant activation of NF-*κ*B transcriptional factor (RELA) and NF-*κ*B related genes, with anti-apoptotic or cell proliferation, tumor initiation and promotion function, such as bcl-2, EGFR, TNF-α, WNT5A and STAT3. Notably, we observed similar mRNA and protein expression patterns of NF-*κ*B in GDFs treated HM supporting further the *in vivo* effect of GDFs on NF-*κ*B activation (Figure 4A and Figure 7A). We also show a significant positive correlation between transcriptional levels of NF-*κ*B and anti-apoptotic bcl-2, oncogenic WNT5A [51], or TNF-α gene, which have been implicated by others in tumor initiation and progression of epithelium [22], supporting further crosstalk between GDFs stimulus-response and tumorigenic processes in HM *via* an NF-*κ*B oncogenic pathway.

We assert that the combination of acid and bile may be more effective in NF-*κ*B related pre-malignant alterations of HM, as compared to neutral bile salts, having demonstrated significantly higher nuclear levels of activated NF-*κ*B (p-p65) and ΔNp63, as well as the most intense nuclear levels of activated STAT3 (p-STAT3), in pre-malignant acidic-bile treated HM. Our *in vivo* findings here are in line with our recent *in vitro* results from HHKs showing that acidic-bile preferentially induces significant transcriptional activation of NF-*κ*B transcriptional factor RELA and NF-*κ*B related genes, such as oncogenic ΔNp63, STAT3 EGFR, and WNT5A, compared to controls. In addition, our data provide evidence that bile refluxate has tumorigenic potential on HM, demonstrated by the *in vivo* effect of DCA to induce premalignant lesions related to molecular alterations, through NF-*κ*B activated pathways.

In conclusion, our novel *in vivo* mouse model shows that the GDFs effect linked to early pre-malignant alterations in murine hypopharyngeal mucosa, is mediated by the NF-*κ*B activated pathway and is related to acidic bile salts and their components CDCA and DCA. Combination of bile and acid contribute significantly to NF-*κ*B activation in treated HM, compared to acid alone, or other factors such as glucose, and induce pre-malignant lesions exhibiting increased cell proliferation rates, as determined by elevated Ki67, CK14 and ΔNp63 protein expression. The combination of bile and acid may particularly be involved in malignant transformation of HM, by activating oncogenic STAT3, previously observed in clinical HNSCCs. We suggest that bile refluxate may be carcinogenic, contributing to ETM, altering cell adhesion molecules, such as E-cadherin and β-catenin. In addition, GDFs induce transcriptional activation of NF-*κ*B factor, RELA(p65), and NF-*κ*B related anti-apoptotic bcl-2, as well as oncogenic EGFR, TNF-α, WNT5A and STAT3 genes in treated murine HM. It is likely true that all hyperplastic/dysplastic lesions (regardless of chosen stimulus) produce oncogenic markers. In this case our profile of markers is specifically linked to histologic lesions produced by bile *vs*. to controls, acknowledging that some markers may be generic to the lesion itself. Although an *in vivo* model may not always correlate with clinical conditions, the negative or reduced effects of acid alone and/or bile salts at neutral pH, compared to acidic bile salts suggest the latter may be especially injurious. One may also suggest that acid reflux alone without duodenal salts lacks high risk properties but pharmacologic acid reduction in the face of gastroduodenal exposure may confer a clinically protective value. In the near future we hope to extend our *in vivo* model to determine the possible additive or synergistic effects of pepsin, tobacco and alcohol on GDFs exposed HM and to investigate the anti-tumor effects of NF-κB inhibition, contributing to hypopharyngeal cancer therapy.

## MATERIALS AND METHODS

### *In vitro* model

#### Generation of murine hypopharyngeal primary cell culture

We generated a murine hypopharyngeal primary cell culture by dissection of hypo-pharyngeal tissue fragments (HTF) from healthy wild-type C57Bl6J mice and dissociated cells from HTF by enzymatic disaggregation as described in supplemental methods 1 (Figure 1A).

#### Bile/acid treatment

Murine hypopharyngeal primary cells (MHPCs) (2nd passage) underwent repeated exposure for 15 min, 3 times per day, for 5 days to (a) acidic bile salts mixture, containing 400 μM of conjugated bile salts mixture (GCA+TCA+GCDCA+TCDCA+GDCA+TDCA, Sigma, St. Louis, MO; Calbiochem, San Diego, CA; USA) at molar concentration (20:3:15:3:6:1) as previously described [17, 29], in DMEM/F12 10% FBS (Gibco^®^, NY, USA), brought to a pH of 4 with 1M HCl, using a pH meter, (b) acidic full growth media (DMEM/F12 10% FBS, brought to pH 4 with 1M HCl) was considered as positive control, and (c) neutral full growth media (DMEM/F12 10% FBS, pH 7.2), was considered as negative control (Supplementary methods 1). The media were removed and replaced with KGM-2 SF (KGM™-2 Keratinocyte Growth Medium-2, Lonza) containing: BPE (Bovine Pit. Extract, hEGF, and antibiotics (penicillin/streptomycin, Invitrogen™Life Technologies, NY, USA), at pH 7.2 until the next treatment.

#### Western blotting

The MHPCs were harvested to perform western blotting analysis in cytoplasmic and nuclear extracts for NF-*κ*B p65 (F-6; Santa Cruz Biotechnology Inc., TX, USA), p-NF-*κ*B (p65 S529) (Invitrogen™), phospho-IKB-α Ser32/36 (5A5; Cell Signaling, EMD Millipore, MA, USA) and bcl-2 (C-2; Santa Cruz), as described in supplementary methods 1. Cytoplasmic and nuclear protein levels were normalized to β-actin C4; Santa Cruz) and histone 1 (AE-4; Santa Cruz), respectively. Protein levels were quantified by Gel imaging system (*BIO-RAD,* CA, USA), and expression levels were estimated by Image Lab 4.1 analysis software 4, BIO-RAD).

### *In vivo* model

#### Animal model

*Mus Musculus,* mouse strain C57BL/6J, was obtained from the Jackson Laboratory (Jax^®^ mice, USA). We followed procedures according to approved protocol 2013-11039/2014-11039, by IACUC (Yale University).

#### Bile/acid treatment

We used a mixture of bile salts (neutral or acidic) at molar concentrations previously described, and considered to be close to “physiologic”, [11, 17, 29, 30]. Additionally, we used two bile acid components, chenodeoxycholic acid (CDCA; Sigma Aldrich^®^, MO, USA) and deoxycholic acid (DCA; Alfa Aesar^®^, MA, USA). DCA had previously been shown to induce DNA damage and apoptotic resistance in Barrett's esophagus *via* NF-*κ*B [8] and CDCA was included in our study, as previously correlated by Sasaki et al., to induce a marked inflammatory infiltrate on laryngeal mucosa, *in vivo* [2]. The GDFs were applied topically to HM, at concentrations and doses, as follows:

Bile/acid fluids (GDFs), of 0.15 ml in volume, were administrated to the upper esophagus and hypopharynx of mice by a plastic feeding tube (20 g), two times per day, for 45 days. Forty female mice, 7 weeks of age were included in our animal model. Animals were separated into 4 experimental and 4 control groups (5 mice/group). Experimental groups were treated by (a) neutral bile salts fluids (10 mmol/l in buffer saline; pH 7.0), containing conjugated bile salts mixture similar to that performed in our *in vitro* study (b) acidic bile salts fluids (10 mmol/l in buffer saline; pH 3 adjusted by 1M HCl), (c) unconjugated deoxycholic acids (DCA) (10 mmol/l, in buffer saline; pH 7.0) and (d) unconjugated chenodeoxycholic acid (CDCA) (10 mmol/l in buffer saline; pH 7.0) (Sigma Aldrich^®^, MO, USA). Control groups included (a) an untreated group of 14 weeks age mice, which was considered as normal control group; (b) an acid alone treated group, without bile salts, (buffer saline; pH 1.5), which was considered as acid control group; (c) a glucose-treated group, which was treated by a glucose solution of high concentration (33 mmol/l in saline, pH 7.0) (Sigma Aldrich^®^, MO, USA) [17], and was considered as a negative control group; as well as (d) a saline-treated group (pH 7.0), which was treated in parallel to the glucose group and was considered as reference group regarding gavage usage.

At the end of the procedures experimental and control animals were euthanatized (IACUC euthanasia policy and guidelines). The euthanized animals were kept on ice for dissection of hypopharyngeal tissue fragments (HTF). The HTF from 4 animals of each group were placed immediately into 10% Neutral buffered formalin (Thermo Fisher Scientific, VA, USA) to be submitted for embedding in paraffin blocks (Yale Pathology Facilities), while the remaining one HTF from each experimental and control groups was immersed in RNA stabilization solution (RNA*later*^®^, Life Technologies, NY, USA) and kept in −80°C, for RNA isolation.

### Tissue examination (H&E staining)

We examined hematoxylin and eosin (H&E) stained 3-4 μm thick tissue sections of formalin fixed and paraffin embedded (FFPE), hypopharyngeal mucosa by light microscopy. Images were captured and analyzed by Aperio CS2, Image Scope software (Leica microsystems, IL, USA). Histopathological alterations were assessed according to criteria (WHO, Ljubljanska) [31, 32] and laboratory mouse histology (Atlas of Laboratory mouse Histology, Claudio J Conti et al, 2004) [33]. Specifically, stratified keratinizing squamous epithelium with a single layer of basal cells characterized normal HM, and was considered as controls. Abnormal hyperplasia was characterized by thickened stratified epithelium with expansion of basal cells into the suprabasal layer without cytologic atypia, considered by some as a pre-cursor for malignant transformation [34, 35]. Thickness of epithelium was measured using the Aperio CS2, Image Scope software (Leica microsystems). In the case of dysplastic epithelium architectural disorder and hyperchromatic or pleiomorphic basal cells expanded in the stratum spinosum. Moderate dysplasia was indicated by characteristic full thickness nuclear hyperchromatism with high degree of basal layer expansion and/or nuclear hyperchromatism with increase of nuclear to cytoplasm ratios, and/or loss of cell polarity into the middle third of the mucosa. (All tissue specimens were examined to exclude histological signs of local treatment toxicity, such as hemorrhagic lesions, ulceration or inflammation)

### Immunohistochemical (IHC) analysis

#### A Chromogenic staining

We performed chromogenic IHC analysis, using immunoperoxidase (DAB peroxidase substrate), for p-NF-*κ*B, in hypopharyngeal tissue sections (HTS) from all experimental and control specimens, in order to identify GDFs-induced NF-*κ*B activation in murine HM*, in vivo*, compared to controls. We used 1:100 dilution of anti-phospho-p65 (rabbit polyclonal anti-phospho-p65 Ser536, AbD Serotec, *BIO-RAD,* CA, USA), to detect protein in the nuclei and/or cytoplasm of basal/parabasal/suprabasal cells of murine hypopharyngeal mucosa. We used positive controls for NF-*κ*B staining, and non-template negative control, in each IHC assay, as recommended by manufacturer. We analyzed the slides, using a light Leica microscope. We captured the images using Aperio CS2. The images were analyzed by Image Scope software (Leica microsystems, IL, USA), which generated algorithm(s) illustrating the mucosal and cellular compartments demonstrating p-NF-*κ*B staining.

#### B Immunofluoresence staining (IF)

We selected at least two tissue specimens from each experimental and control group, including specimens with premalignant lesions, in order to identify NF-*κ*B activation and additional molecular alterations related to malignant transformation of HM. We performed IF staining for p-NF-*κ*B (S536), Ki67, ΔNp63, CK14, E-cadherin, β-catenin and p-STAT3 (Tyr705), in serial HTS of the selected specimens, using DyLight^®^488 for green or DyLight^®^549 for red (Vector Labs, CA, USA). Specifically, we used 1:100 anti-phospho-p65 (S536) (#AHP1342, AbD Serotec, BIO-RAD, CA, USA) to detect p-NF-κB as described in chromogenic IHC analysis; 1:300 of anti-ΔNp63 (#M7247, mouse monoclonal anti-p63 antibody, clone 4A4, Dako, Aglilent Technologies, Denmark) to detect protein in the nuclei of basal/parabasal cells of HM; 1:200 dilution of anti-Ki67 (rabbit mAb, SP6, Thermo ScientificTM Lab Vision, UK) to detect protein in nuclei of basal/suprabasal cells of HM; 1:80 dilution of anti-E-cadherin (mouse mAb, NCH-38, Dako, CA, USA), and 1:100 dilution of anti-β-catenin (β-catenin rabbit mAb, E247], abcam^®^, MA, USA) to detect cell-cell adhesion molecules immunoreactivity in murine hypopharyngeal mucosa; and 1:100 anti-cytokeratin 14 (#ab7800, mouse mAb, LL002, abcam^®^, MA, USA) to detect protein localization in the basal layer or upper layers of HM; 1:100 p-STAT3 (Tyr705) (rabbit mAb, D3A7 XP^®^, Cell Signaling Technology, Inc., MA, USA) to detect protein nuclear localization of basal/suprabasal cells of HM. We used anti-rabbit or anti-mouse secondary DyLight^®^488 for p-NF-κB, Ki67, CK14 and p-STAT3, and DyLight^®^549 for ΔNp63, E-cadherin and β-catenin and DAPI to distinguish the nuclei (DyLight^®^488 for green or DyLight^®^549 for red; Vector Labs, USA). At the end of IF staining the slides were examined microscopically and their images were captured for analysis (Zeiss fluorescence microscope, AxionVision system; Carl Zeiss microscopy, NY, USA).

### AQUA analysis

We performed an automated quantitative analysis (AQUA), in order to measure the protein expression levels of p-NF-*κ*B, Ki67, ΔNp63, CK14, E-cadherin, β-catenin and p-STAT3, in treated HM relative to untreated controls. In brief, images of HM tissue sections (derived from each experimental and control group of animals) were captured and analyzed using PM-2000 image workstation and HistoRX^®^ software, as previously described [36]. For each whole tissue section, areas of HM were selected, while compartments of submucosa were excluded, using AQUA software. The signal intensity of target antigen was acquired using DyLight^®^488 (similar to FITC) signal or DYLight549 (similar to Cy3) signal, relative to tumor mask. The signal intensity of nuclei was acquired using DAPI signal. Maturing squamous cells that were losing their nuclei were counted as negative cells. Aqua scores within nucleus and cytoplasm (or membrane) were calculated dividing the signal intensity by the area of the specified compartment.

### Quantitative real time PCR

We performed real time qPCR analysis (Bio-Rad real-time thermal cycler CFX96^TM^), in order to determine the transcriptional levels of oncogenic NF-*κ*B related pathways, as we had previously characterized GDRD-induced and hypopharyngeal cancer related phenotypes in our *in vitro* study on HHKs [17], in GDFs treated-HM and untreated controls. We isolated total RNA from representative murine HTS of GDRD-treated groups, acidic-bile, neutral-bile, DCA and CDCA, and normal-untreated group, using RNeasy mini kit (Qiagen^®^, KY, USA), in order to perform real time qPCR. RNA quality was determined by absorption ratios at 260/280 nm (>2.0) and concentration ratios by absorption at 260 nm, using a NanoDropTM 1000 spectrophotometer (Thermo Scientific). Reverse transcription to cDNA was performed using Whole Transcriptome kit (Qiagen^®^, KY, USA), following the manufacturer's instructions. The real time qPCR analysis (Bio-Rad real-time thermal cycler CFX96TM) was performed using specific primers for mouse genome as indicated in Table 1 and iQTM SYBR^®^ Green Supermix (BIO-RAD, CA, USA). Specifically target genes included NF-κB (RELA), bcl-2, EGFR, TNF-α, WNT5A and STAT3 while GAPDH was used as a reference control gene (QuantiTect^®^ primers assay, Qiagen^®^, KY, USA), in order to identify transcriptional expression levels of GDRD-induced and NF-κB related genes that we identified in our prior *in vitro* study [17]. PCR assays were performed in 96 well plates and each sample was assayed in triplicate. Q-real time PCR data were analyzed by CFX96TM software (Bio Rad, CA, USA). Relative expression levels of experimental (acidic-bile, neutral-bile, DCA and CDCA treatment) to normal (untreated) mRNAs, were estimated by CFX96^TM^ (Bio Rad) software, for each specific gene.

### Statistical analysis

We performed a statistical analysis using Graph Pad Prism 6 software. Data obtained from AQUA and real time qPCR analysis were calculated and analyzed by HistoRX^®^ (HistoRX^®^ Inc. CT, USA) and CFX96^TM^ (Bio Rad) software, respectively. To obtain differences of AQUA scores, thickness of mucosa or mRNA expression values between experimental and control groups we performed comparisons using ONE-WAY ANOVA, non-parametric Kruskal-Wallis or Friedman test and Dunn's multiple comparison test (significance was defined as *P*-values < 0.05). The correlation coefficient (*r*) between protein or mRNA expression levels of different groups was estimated *by Pearson* correlation (significance *P* values < 0.05). A *Z*-test was used to estimate significance of protein or gene expression differences between experimental and control groups that were estimated by Image Lab 4.1 analysis software 4, or CFX96TM (*BIO-RAD,* CA, USA).

## SUPPLEMENTARY TABLE, FIGURES AND VIDEO


