# Supplementary material for: Gastro-duodenal fluid induced nuclear factor-κappaB activation and early pre-malignant alterations in murine hypopharyngeal mucosa
Source: Oncotarget. 2016 Jan 6;7(5):5892–908. doi: 10.18632/oncotarget.6824 (PMC4868729; doi:10.18632/oncotarget.6824)
Supplement: Supplementary file 1 [file oncotarget-07-5892-s001.pdf]

Gastro-duodenal fluid induced Nuclear Factor-*κappa*B activation and early pre-malignant alterations in murine hypopharyngeal mucosa

Supplementary Material

Supplement methods 1:
In vitro model

Generation of murine hypopharyngeal primary cell culture*. Dissection of mouse hypo-pharyngeal tissue.* Tissue fragments were harvested from the hypo-pharynx of 8 healthy wild type mice (*Mus Musculus*, mouse, strain C57BL/6Jm age 16-21 weeks; Jax®Mice, USA). Healthy animals were euthanized, using carbon dioxide, following the new IACUC euthanasia policy, guidelines and procedures [CO_2 100%-1-3 gas/min in 10 L chamber-total time 2-3 min] and kept on ice. Normal tissue characterization included clinical examination of tissue before dissection. Hypopharyngeal tissue fragments (HTF) were rinsed four times with basal medium serum free and calcium free (KGM™-2 Keratinocyte Growth Medium-2; Lonza, Gibco®, NY, USA) containing: BPE (Bovine Pit. Extract; Lonza, Gibco®, NY, USA), hEGF (Lonza, Gibco®, NY, USA), and antibiotics (penicillin/streptomycin, Invitrogen™, Life Technologies, NY, USA; normocin/fungin, InvivoGen, CA, USA) and put immediately on ice. One representative piece of HTF was immersed in 10% neutral buffered formalin (Sigma Aldrich®, MO, USA) and was submitted to Pathology for phenotyping (YPTS, Yale). The remaining tissues were enzymatically disaggregated.

Dissociation of cells from HTF (enzymatic disaggregation*)*. Working under laminar flow we cut each piece of tissue into 3-4 mm fragments, using a fine scalpel (on ice). The fragments were incubated in a 1:1 solution of KGM and 0.25% trypsin (GIBCO-BRL; CA,USA) in a small culture dish for 12 h at 4°C. After initial enzymatic incubation we washed the cells with 5 ml of phosphate-buffered saline (PBS) containing 2% FBS (Gibco®, NY, USA) followed by vigorous shaking for several minutes. The residual tissues were incubated with additional 0.05% trypsin at 37°C for 3 min. The remaining epithelial

fragments were removed from the tube, and the shaking was repeated with fresh PBS-2% FBS in another tube. The remaining tissue was removed, and cell suspensions were pooled and pelleted by centrifugation at 400 × g, for 5 min. The cell pellets were re-suspended in 2 ml basal medium serum free supplemented by calcium, BPE, hEGF and antibiotics (penicillin/streptomycin, Gibco®, NY, USA; normocin/fungin, InvivoGen, CA, USA) and were plated in 25cm^2 Tflasks (Mouse Hypopharyngeal Cell Culture Extra-cellular Matrix T25 Flasks; Celprogen, CA, USA) culturing at 37°C in humidified air and 5% CO_2. We added 2 ml warm medium every 48h for total 7 days. After 7 days we changed the medium without Normocin/Fungin. The cells began to proliferate after 10 days reaching 50% confluence after 5-7 days and 70% after 15 days and then were passed to fresh dishes (Fig. 1A).

Bile/acid treatment. We exposed murine hypopharyngeal primary cells (MHPCs) repetitively to an acidic bile salts mixture, in parallel with neutral and acidic controls, according to our prior *in vitro* study (ref 17). At the end of experimental procedures media were removed and cell extracts were analysed by western blotting.

Western blotting: Total cytoplasmic and nuclear protein concentrations of treated-MHPCs extracts (NE-PER Nuclear & Cytoplasmic Extraction including protease inhibitors; Thermo Scientific Pierce, MA, USA) were determined using the BCA-200 Protein Assay kit (Pierce). Specifically, 10-30 µg of cytoplasmic and nuclear protein extracts were mixed with SDS-PAGE Laemmli sample buffer (*BIO-RAD*), heated at 70°C for 10 min, and were separated using 15% Mini-PROTEAN TGX™ Tris/Glycine pre-cast gels, at 220 V for 1 h. We used Precision Plus Protein™ Standards (Kaleidoscope™, CA, USA) providing a 10-band ladder (250-10kD) (BIO-RAD, CA, USA). Proteins were transferred onto a 0.45 µm nitrocellulose membrane, using Trans Blot® Turbo™ transfer system, blocked in 5% bovine serum albumin (BSA) (Sigma-Aldrich®, MO, USA), for 1 h, and were incubated with NF-*κ*B (p65, p-p65), phospho-IKB-α Ser32/36 and bcl-2 primary antibodies that were diluted in blocking

buffer (5% BSA), overnight at 4°C. Membranes were washed in TBST and incubated for 1:30 h with goat anti-rabbit or anti-mouse horseradish peroxidase (HRP) conjugated secondary antibodies (EMD Millipore, MA, USA) at 1:5,000 and chemiluminescence was determined using ECL detection system (Clarity Western ECL Substrate, *BIO-RAD,* CA, USA). Membranes were stripped using Restore stripping buffer (Pierce) and were reported with β-actin (C4; Santa Cruz) and histone 1 (AE-4; Santa Cruz), for cytoplasmic and nuclear extracts normalization, respectively (Gel imaging system; *BIO-RAD,* CA, USA).
